# 5hmC-profiles in Puerto Rican Hispanic/Latino men with aggressive prostate cancer

**DOI:** 10.3389/fonc.2025.1541878

**Published:** 2025-04-08

**Authors:** Manishkumar S. Patel, Mousa Almubarak, Jaime Matta, Carmen Ortiz-Sanchez, Jarline Encarnacion, Gilberto Ruiz-Deya, Julie Dutil, Jasreman Dhillon, Kosj Yamoah, Anders Berglund, Hyun Park, Deepak Kilari, Yoganand Balagurunathan, Liang Wang, Jong Y. Park

**Affiliations:** ^1^ Department of Tumor Microenvironment and Metastasis, H. Lee Moffitt Cancer Center and Research Institute, Tampa, FL, United States; ^2^ Department of Basic Sciences, Ponce Research Institute, Ponce Health Sciences University-School of Medicine, Ponce, Puerto Rico; ^3^ Department of Pathology, H. Lee Moffitt Cancer Center and Research Institute, Tampa, FL, United States; ^4^ Department of Radiation Oncology, H. Lee Moffitt Cancer Center and Research Institute, Tampa, FL, United States; ^5^ Department of Biostatistics and Bioinformatics, H. Lee Moffitt Cancer Center and Research Institute, Tampa, FL, United States; ^6^ Department of Cancer Epidemiology, H. Lee Moffitt Cancer Center and Research Institute, Tampa, FL, United States; ^7^ Division of Oncology, Medical College of Wisconsin, Milwaukee, WI, United States; ^8^ Department of Machine Learning, H. Lee Moffitt Cancer Center and Research Institute, Tampa, FL, United States

**Keywords:** 5hmC, prostate cancer, DNA methylation, Puerto Rican Hispanic/Latino, aggressiveness

## Abstract

**Introduction:**

Puerto Rican (PR) Hispanic/Latino (H/L) men are an understudied population that has the highest prostate cancer (PCa) specific mortality among other Hispanic populations. Little information is known about the higher mortality in PR H/L men. It is thought that epigenetic changes in key genes may play a critical role in aggressive tumors.

**Methods:**

We aimed to identify key 5-hydroxymethylcytosine (5hmC) changes in PR H/L men with aggressive PCa. We performed sequencing analysis using the 5hmC-enriched DNA from 22 prostate tumors and 24 adjacent normal FFPE samples.

**Results:**

We identified 808 differentially methylated genes (DMGs) in tumors compared to adjacent normal tissues. These genes suggest key mechanisms, including upregulated signatures of negative Androgen Receptor (AR) regulation, Wnt/β-catenin pathway activation, and downregulation of tumor suppressor genes. Pathway analysis of DMGs demonstrated that DNA repair pathway was most upregulated in tumors. Since 5hmC abundance positively correlates with gene expression levels, we further investigated 808 DMGs in TCGA PCa gene expression data. Further, we identified 59 DMGs with significant gene expression changes in the same direction. Additionally, we identified 111 aggressiveness-related DMGs, of which, two hypomethylated genes (*CCDC122*, *NUDT15*) and four hypermethylated genes (*PVT1*, *RPL30*, *TRMT12*, *UBR5*) were found to be altered at transcriptomic level in a concordant manner in PR H/L PCa patients. Aberrant 5hmC and GE changes in these six genes were also associated with progression-free survival in the mixed PCa population.

**Discussion:**

The 5hmC modifications and associated gene expression changes in these six genes could be linked to the highest prostate cancer (PCa)-specific mortality in PR H/L men. In conclusion, our study identified 59 DMGs showing concordant epigenetic and transcriptomic changes in tumor tissues and 111 DMGs showing association with aggressive PCa among PR H/L men. Our findings have significant implications for understanding these key genes’ molecular mechanisms, which may drive PCa progression and mortality in this population. This will help in developing potential biomarkers or therapeutic targets for personalized treatment strategies in this high-risk subgroup. Future research will explore how these genes contribute to PCa-specific mortality through molecular analyses, with plans to validate them in a larger validation cohort.

## Introduction

In 2024, 299,010 new prostate cancer (PCa) cases and 35,250 PCa-specific deaths are anticipated in the US (1). The lifetime risk of PCa in US men is approximately 12.5% (2). PCa-specific mortality (PCSM) rates have been found to vary among different racial/ethnic groups in the US, especially in certain Hispanic subgroups as compared to non-Hispanic Whites (NHWs) and non-Hispanic Blacks (NHBs) (3). This study combined all Hispanic subgroups into one broad group including Mexican Americans, Caribbeans, Puerto Ricans, and South Americans. However, among different Hispanic/Latino (H/L) subgroups, Puerto Rican men showed significantly higher PCSM rates than other Hispanic groups and NHBs (3) (**Table 1**). Indeed, PCa is the most common cancer case and cancer-specific death in Puerto Rico (4). According to the 2019 PR Cancer Registry data, PCa is the leading cancer type in terms of incidence 137.3/100,000) and mortality (16.2/100,000) in Puerto Rican (PR) H/L men (5). Despite the high level of PCa vulnerability in this population, the underlying causes of the high mortality rate in this group are still unclear.

**Table 1:** incidence and mortality rates for prostate cancer in Latin countries.

Country	Incidence rate (per 100000 persons per year)	Mortality rate (per 100000 persons per year)
Puerto Rico (5)	137.3	16.2
Mexico (64)	42.2	10.6
Peru (65, 66)	40.1	10.5
Brazil (67)	81.5	14.5
Chile (66)	62.3	15.1

PCa is a complex disease that is mediated by the accumulation of genetic and epigenetic aberrations, such as differential expression of oncogenes and tumor suppressor genes (6). Differential DNA methylation can influence carcinogenesis and disease progression (7). Epigenetic changes such as DNA methylation (5-hydroxymethylcytosines (5hmC) and 5-methylcytosines (5mC)) are important mechanisms responsible for transcription regulation and ultimately functional implications to drive aggressive pathology of PCa (8, 9). Zhao et al. reported differential 5mC changes during PCa progression at putative regulatory regions. Indeed, the most common molecular events in PCa are DNA methylation dysregulation. Among these epigenetic changes, some specific changes may be associated with poor outcomes, including PCSM, metastasis, and recurrence (10). The Cancer Genome Atlas (TCGA) study found associations between gene expression and methylation profiles. This study suggested that epigenetic changes define distinct molecular subtypes of PCa (11). The role of DNA methylation in promoter regions has been investigated numerously, and many differentially methylated genes have been related to gene silencing of tumor suppressor genes in PCa and with poor outcomes (7, 12–14).

In addition to commonly known 5-methylcytosine methylation in the genome, 5-hydroxymethylcytosines (5hmC) are also reported. These 5hmCs are created by oxidation of common 5-methylcytosine methylation by ten-eleven translocation (TET) enzymes (15). Several studies reported a regulatory role of 5hmC in gene expression (8, 16). Like common 5-methylcytosine methylation, locations of 5hmC are in gene bodies, promoters, and enhancers, which are transcriptionally active regions (17). However, unlike 5mC, the functional role of 5hmc is overexpression of gene regulation (18). Therefore, 5hmCs were suggested as a new class of epigenetic biomarkers for various cancers, including PCa (8, 19, 20).

Notably, 5hmC modification is predominant in gene bodies and can be a better marker in echoing gene expression than gene body 5mC (21). Also, around 33% of 5hmC peaks are in tissue-specific differentially methylated regions potentially affecting tissue-specific functional gene expression in the same direction (21, 22). 5hmC DNA methylation also has an essential tissue-specific function in epigenomic activation in PCa and it was identified as a potential biomarker of aggressive PCa (8). This study identified that 5hmC levels of genes such as EZH2 and TOP2A associated with poor survival in PCa. Transcriptomic levels of these genes were shown to be hallmark of aggressive PCa (23). This depicts that 5hmC patterns can find epigenomic activation of driver genes associated with aggressive PCa. However, it is not known why PR H/L men show high PCa-specific mortality. Since differential DNA methylation may influence racial disparities in PCa, there is a need to investigate 5hmC profiles to evaluate potential PR-specific methylated genes associated with poor prognosis. To identify promising 5hmC biomarkers for aggressive PCa in PR men, we applied the 5hmC-Seal technology (24) to examine methylation changes in PCa tissues from PR men. Our results suggested that differential 5hmC changes in a group of candidate genes are associated with aggressiveness and potentially contribute to cancer disparity.

## Methods

### IRB approval and tissue sample collection

Two Institutional Review Boards, the Moffitt Cancer Center (Protocol no. Pro00048100) and the Ponce Health Sciences University (PHSU) (Protocol no. 1909021277A001), approved this study. All study participants signed an Informed Consent. We obtained 88 formalin-fixed paraffin-embedded (FFPE) prostate tumor and adjacent non-involved pair samples from the Puerto Rico Biobank (PRBB), a U54 PHSU-MCC PACHE Partnership core facility. All prostate tissues were obtained from prostate cancer patients who were treated surgically. We excluded the patients with metastasis, and who were treated by radiation. Based on Gleason scores and following the 2023 National Comprehensive Cancer Network (NCCN) guidelines for prostate cancers, tumors from study participants were classified as either aggressive or indolent.

### Gene expression

mRNA transcript quantification was done using the Human Exon 1.0 ST microarray (Thermo-Fisher, Carlsbad, CA, USA) at the Genomic Core at Moffitt Cancer Center. RNA was extracted from the FFPE blocks using macro-dissection from 88 PR H/L prostate cancer patients. The microarray measures 46,050 RNA transcripts. The SCAN (25) algorithm was used to preprocess and normalize the transcriptomic data resulting in log2 gene expression. Decipher, a 22-marker prognostic gene-expression score, was determined from the Decipher Prostate cancer classifier assay (25, 26). We used the following cut-off values for Decipher score, 0.0 – 0.45, low risk for metastasis, 0.46 – 0.60, intermediate risk, and 0.61 – 1.00, high risk.

### DNA extraction and quality control

Genomic DNA samples were obtained from the FFPE prostate tumor tissues as described in the manufacturer’s instructions (QIAamp DNA FFPE Tissue kit, Qiagen, Germantown, MD). DNA was extracted from the marked tumor area on the H&E slides by the pathologist (J.D.) from 46 (22 tumor and 24 adjacent normal) tissues from 88 PR H/L patients. DNA quality was tested with DNA integrity numbers (DINs) using Tapestation (Agilent Technologies). The mean DIN score was 4.07 with range 1.7-6.0. We used the cut off >2.5. DNA was quantified using Qubit 2.0 fluorometer (Life Technologies) with Qubit dsDNA HS Assay Kit (Life Technologies).

### 5hmC library preparation

We used 7-50 ng of genomic DNA as starting material. Briefly, DNA polishing (at 37°C for 30 min) and enzymatic fragmentation (at 37°C for 5 min.) were carried out using NGS FFPE DNA polishing kit (KAPA/Roche, USA) and DNA fragmentation kit (KAPA/Roche, USA) as per manufacturer’s instructions. After fragmentation, the DNA sample was end-repaired and A-tailed using KAPA/Roche Hyper Prep Kit PCR-Free as per manufacturer’s instructions. End-repaired DNA was ligated with adapters (5 NEBNext Multiplex Oligos, Illumina), processed further for USER enzyme digestion, and purified. After digestion, DNA was enriched by labeling and capturing as described previously (24). The enriched DNA was used for qPCR (4 μl) and library amplification (20 μl). Fold change was used to describe the relative enrichment and it was calculated by Δ-Δ Ct formula (2^(–ΔΔCt)^) = (ΔCt Sample) – (ΔCt control). The 5hmC DNA was amplified using universal primer (New England Biolabs, USA), index primer (New England Biolabs, USA) and HiFi HotStart ReadyMix (KAPA/Roche). Further, purified libraries were quantified using the Quantus fluorometer instrument (Promega) and the QuantiFluor^®^ ONE dsDNA kit (Promega). The quality of the libraries was assessed using the TapeStation system. The library size distribution within the range of 200-600 bp across all samples was evaluated, indicating consistent and high-quality libraries. Next, Single-end 75 bp sequencing was performed on an Illumina NextSeq 500. 22 prostate tumors and 24 adjacent normal FFPE samples were sequenced after 5hmC enrichment.

### Sequencing data processing

FastQC was used to evaluate raw read quality (27). Reads were aligned to human genome build hg38 from Ensembl (https://ftp.ensembl.org/pub/release-111/fasta/homo_sapiens/dna/Homo_sapiens.GRCh38.dna.primary_assembly.fa.gz) using bowtie2 v2.5.1 (28) and sorted and indexed using samtools v1.17 (29). Further, duplicates were removed from mapped reads using Picard (30), and, raw read counts per gene were generated using the feature Counts tool from package subread (31). Using Principal Component Analysis (PCA), we checked samples for outliers. We evaluated samples based on their position relative to the principal components (PCs), specifically looking for samples that deviated significantly from most of the dataset. We did not exclude any samples as outliers based on the PCA plot.

### Differentially methylated genes and pathway analysis

Differentially methylated genes were identified using the DESeq2 package (32). All samples were normalized using Deseq2 internal normalization and further compared in unpaired manner with multiple-hypothesis testing as all tumors and adjacent normals were not paired. Genes with |log2 fold-change| >0.4 and adjusted p-value<0.05 were considered differentially methylated. Fold change was used to describe the relative enrichment and it was calculated by Δ-Δ Ct formula (2(–ΔΔCt)) = (ΔCt Sample) – (ΔCt control). Pathway analysis was performed using GSEA (33). Top pathways were selected based on p_adj_<0.05. An enhanced Volcano package was used to prepare volcano plot. The cluster Profiler package (34) performed pathway analysis and visualized functional profiles of differentially methylated genes.

### Differentially expressed genes analysis-TCGA

IlluminaHiSeq pancan normalized prostate cancer gene expression (N=549) data was downloaded from TCGA Hub. From a total of 549 samples, 52 samples were normal solid tissue biopsies and 497 were primary tumors. To identify differentially expressed genes, we calculated ΔGE (Δ gene expression- differences in GE between tumor and adjacent normal samples) and filtered for genes having ≥ 1 or ≤ -1 value for ΔGE and adjusted p-value<0.05.

### Integration with 5hmC and gene expression changes in PCa patients from mixed origin

5hmC DNA methylation and gene expression values were integrated for DMGs and differentially expressed genes. Log2FC was used for 5hmC and ΔGE values were utilized for TCGA gene expression data. Significantly hypo- or hypermethylated genes showing the same direction of alteration as previously published 5hmC data or gene expression were plotted on the same plot using the ggplot2 package in R (35).

### Risk analysis for 5hmC and Gene expression profile in PR H/L men

Gleason score (GS) was used as a cut-off to identify DMGs in aggressive PCa patients (GS- 7 (3 + 4) or less, Non-aggressive; GS- 7 (4 + 3) and above, Aggressive). For gene expression profile, we used 21 aggressive and unpaired 65 indolent tumors from 88 PR H/L patients. Unpaired two-sided Wilcoxon rank sum test from the ‘stats’ package was used to calculate significantly different 5hmC or gene expression changes in aggressive vs indolent tumors. Pheatmap package (36) was used to create heatmap plots and the ggplot2 package (35) was used to create boxplots.

### cfDNA 5hmC sequencing and survival analysis in advanced PCa patients

We performed 5hmc enrichment and sequencing library preparation as the methods had been previously published (37). In brief, cell-free DNAs (cfDNAs) were extracted from 0.4 – 1.0ml of platelets-poor plasma using QIAamp DNA Blood Mini Kit (Qiagen). The cfDNA yield was quantified using the Qubit and stored at -80°C until use. As described above, 5-10ng of cfDNA was used for 5hmC enrichment and library preparation.

### Survival analysis

Survival analysis was performed for 5hmC data generated from cfDNA (N=55) and gene expression data downloaded from TCGA for prostate cancer patients (N=497). For the association study, clinical data for TCGA dataset was also downloaded from GDC (https://xenabrowser.net). We used Kaplan-Meier survival analysis (lower level = below the median and higher level = above the median) to analyze the association of 5hmC or Gene expression levels with progression-free survival (PFS) as the endpoint. Association with PFS was done using the ‘survival’ package (38), and graphical representations were created using the ‘pheatmap’, ‘survminer’ and ‘ggplot2’ packages. P<0.05 was considered significant. All statistical analyses were performed in R (v4.3.1).

### Data download

Raw 5hmC-seq data from 51 localized and 7 adjacent normal prostate samples were obtained from European Genome-Phenome (https://ega-archive.org/datasets/EGAD00001008462; Study ID: EGAS00001004942). Gene expression data for prostate cancer samples were downloaded from TCGA (https://tcga-xena-hub.s3.us-east-1.amazonaws.com/download/TCGA.PRAD.sampleMap%2FHiSeqV2_PANCAN.gz).

## Results

### Demographic and clinicopathological characteristics of study group

**Figure 1:**
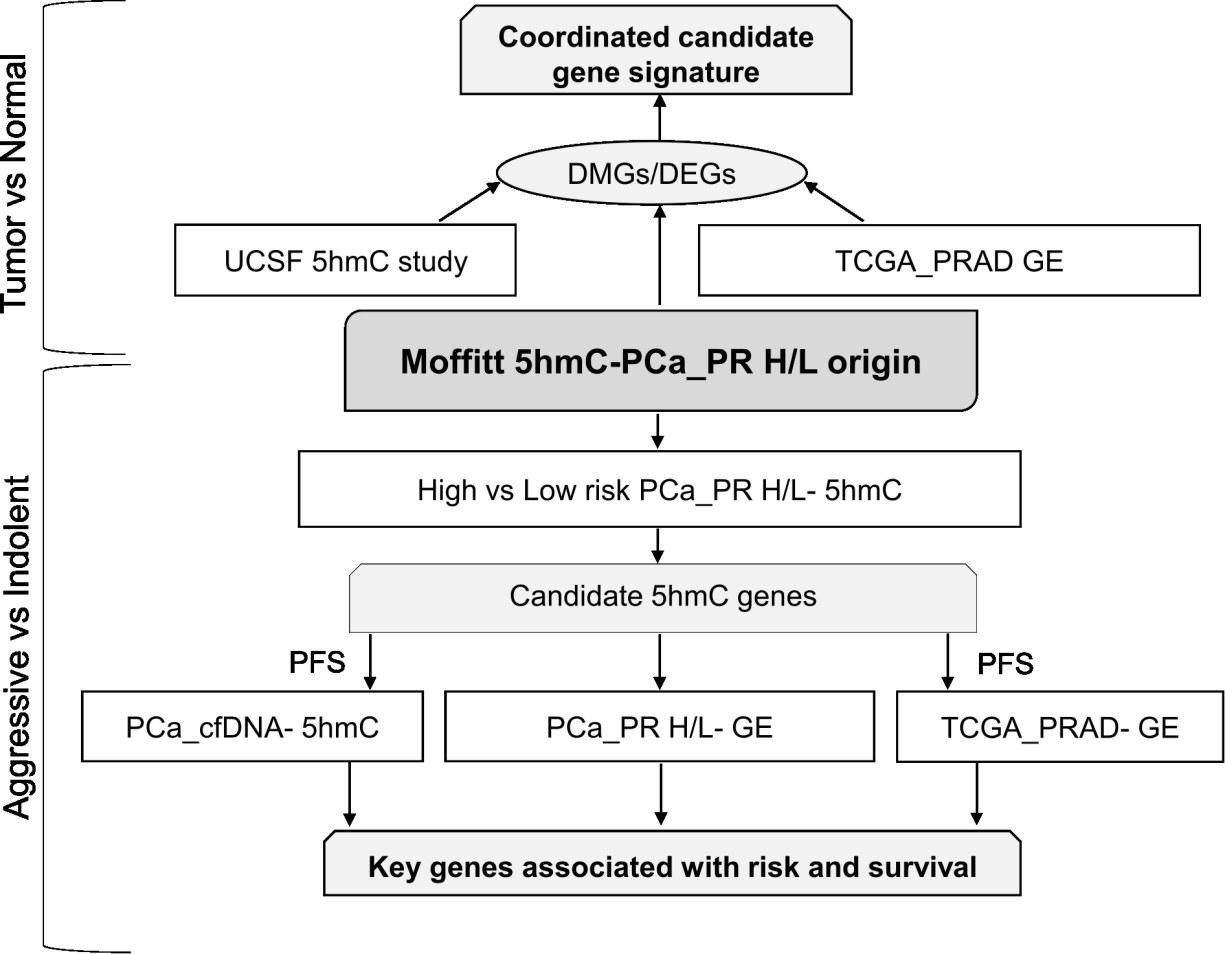
Study workflow. DEGs, Differentially expressed genes; DMGs, Differentially Methylated Genes; GE, Gene Expression; PFS, Progression free survival.

The Puerto Rican population is a genetically admixed with an ancestry structure composed primarily of European, African and Indigenous American ancestries. In a study of 49 PHR H/L PCa patients, the average ancestry was European (65.8%), African (21.9%), and Indigenous American (12.3%) (39). The mean age at diagnosis for PR H/L men with PCa was 62.8 years (**Table 2**). Seventy-six percent of all patients (n = 67) had a low Gleason score (6 or 7 (3 + 4)) and were classified as a low-risk group while 24% of all patients (n = 22) had a high Gleason score (7 (4 + 3) or 8−10) and were classified as a high-risk group. As expected, a significantly different distribution in the clinical stage was detected between the two groups (p=0.02). There were no statistically significant differences between the two groups regarding prostate-specific antigen (PSA) levels. The study workflow was divided into two parts (**Figure 1**). The first part involves a comparison of tumor tissues with adjacent normal controls in PR H/L men and further integration with previously published 5hmC and gene expression datasets of PCa patients from mixed origin. The second part involves risk analysis in PR H/L men with PCa to discover significantly different methylated genes with concordant transcriptomics signatures associated with PCa aggressiveness in PR H/L men. We also validated the DMGs by testing their 5hmC and gene expression levels association with poor survival in PCa patients of mixed origin.

**Table 2:** Clinicopathological Characteristics of Puerto Rican prostate cancer patients.

Patients Clinical Characteristics (n=88)					
		Total (88)	Aggressive (22)	Indolent (66)	P value
Race	White	78	18	60	0.48
	Black	7	3	4	
	Others	3	1	2	
Stage	T1 or T2	72	16	56	0.02
	T3	13	7	6	
Gleason score	6	42	0	42	
	7 (3 + 4)	25	0	25	
	7 (4 + 3)	12	12	0	
	8-9	9	9	0	
Age		62.8 ± 6.67	64.8 ± 5.5	60.8 ± 8.3	0.07
PSA		6.70 ± 4.3	7.51 ± 4.8	6.96 ± 5.5	0.73
Decipher					0.21
	0.0-0.45	39	11	28	
	0.46-0.60	19	3	16	
	0.61-1.0	22	9	13	
Family history		12%	13%	12%	0.91

### Differentially methylated genes in PCa tumors from PR H/L men

**Figure 2:**
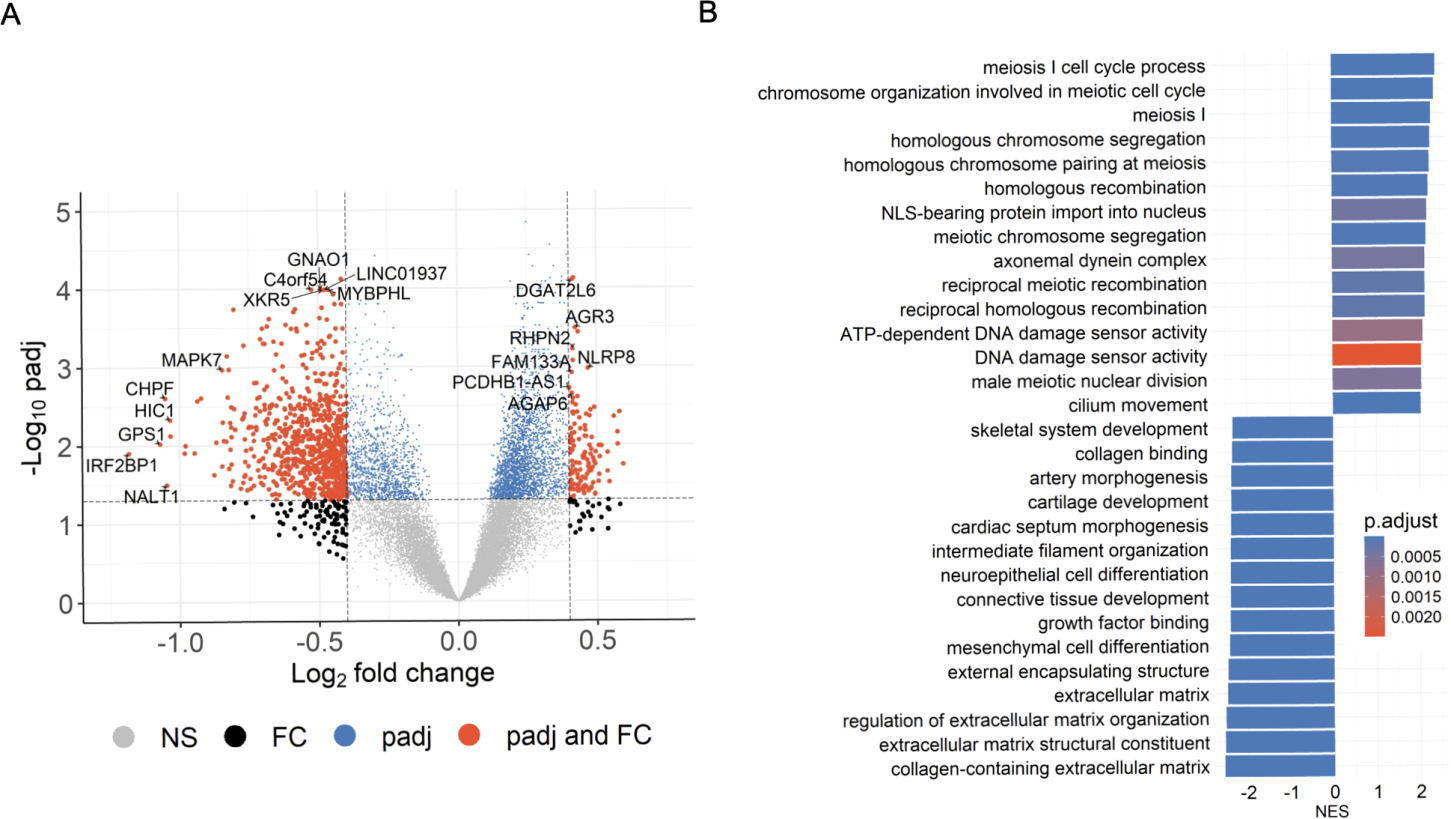
Significant DMGs associated with DNA damage and cell-cycle related pathways in PCa patients of PR H/L origin. **(A)** The volcano plot indicates DMGs in tumor samples (n=22) compared to adjacent normal (n=24); padj<0.05. **(B)** GSEA plot showing top 30 pathways significantly (padj<0.05) altered in tumor tissues compared to adjacent normal tissues. The color intensity represents level of significance.

To identify differentially methylated genes in PR H/L men, we first compared normalized read counts from 22 tumor samples with 24 adjacent normal samples in an unpaired manner, and identified 808 DMGs (FDR<0.05, log2FC>|0.4|) (**Figure 2A**, **Supplementary Table 1**). The most noticeable DMGs included hypermethylated genes (*AGR3, FAM13A, NLRP8, AGAP6, RHPN2, DGAT2L6*) and hypomethylated genes (*IRF2BP1, GPS1, NALT1, HIC1, MAPK7, XKR5, MYBPHL* and *GNAO1*). Since these DMGs may play a key role in PCa, we performed pathway analysis to reveal the biological pathways involved in PCa biology for PR H/L men. This analysis showed that cell cycle, meiosis, cell division, and DNA repair-related pathways were most upregulated in tumor samples compared to adjacent normal samples (**Figure 2B**, **Supplementary Table 2**). This indicates that tumors were highly dysregulated with a lack of apoptotic genes and pathways typically important for regulating growth and survival.

### 5hmC and gene expression signature in PCa

**Figure 3:**
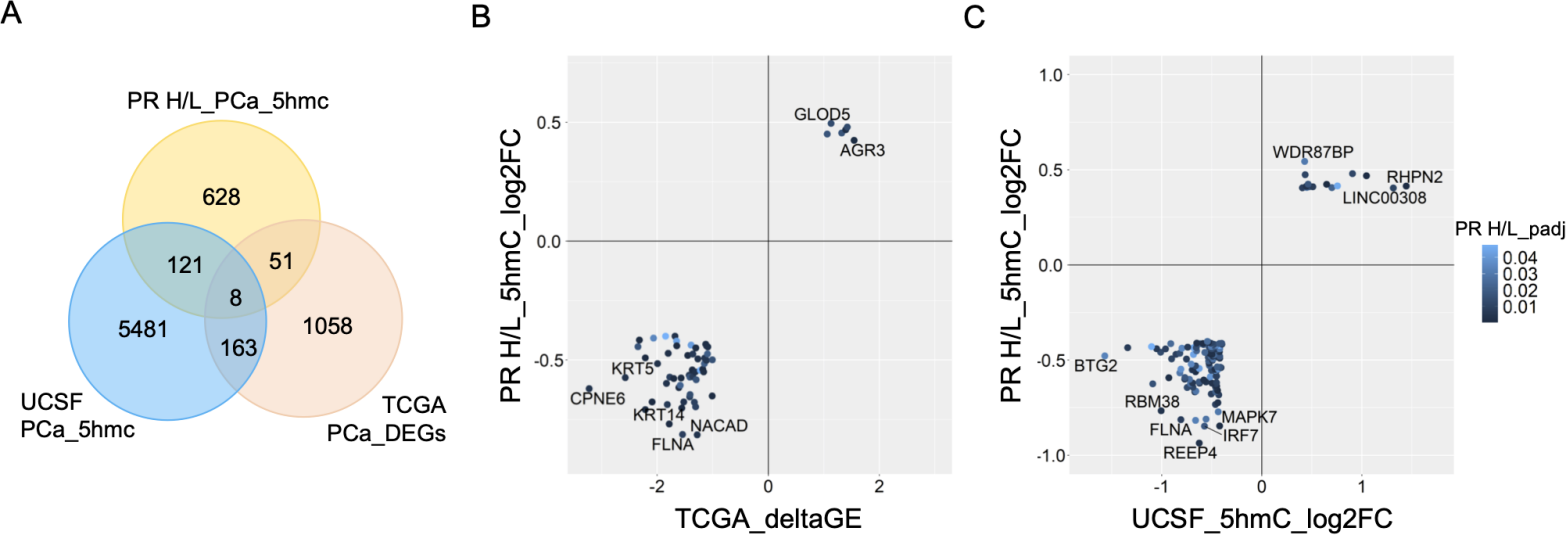
Integration of 5hmC candidate genes from PR H/L PCa patients with UCSF 5hmC and GE dataset from PCa patients of mixed origin. **(A)** Van diagram showing overlapping candidate genes with UCSF 5hmC and TCGA GE datasets (padj<0.05). **(B)** GE (TCGA PCa dataset) and **(C)** 5hmC (UCSF study on PCa patients) changes in the same direction as PR H/L men.

Since 5hmC abundance is directly correlated with gene expression levels, we investigated 808 DMGs’ expression changes in the TCGA prostate cancer dataset. This analysis identified 59 common DMGs (80.1%, (FDR<0.05, ΔGE>|1|) with significant 5hmC and gene expression changes in the same direction (**Figures 3A, B**). This same direction epigenetic and transcriptomic changes (59 genes) in PR H/L men include tumor suppressor genes such as *DKK3* and *PRDM8* with downregulated 5hmC and GE (gene expression) levels (**Supplementary Table 3A**). We also examined the shared 5hmC candidate genes between PCa patients of the PR H/L population and mixed origin. To identify DMGs in the population with mixed origin, we performed 5hmC methylation analysis in 51 localized PCa and 7 normal samples retrieved from the previously published study dataset (8). This analysis showed 129 DMGs shared between two populations (**Figures 3A, C**, **Supplementary Table 3B**). We also observed that the previous 5hmC study showed 171 genes with the same directional gene expression signature in the TCGA dataset. Eight DMGs in PR H/L men have the same direction changes as the other two datasets. Importantly, we found 628 potentially unique 5hmC genes in PR H/L men with PCa (**Supplementary Table 3C**). These unique differentially methylated genes in PR H/L men include hypomethylated genes such as *IRF2BP1*, *HIC1*, *NALT1*, *MAPK11* and hypermethylated genes such as *CDC25C*, *FLT3, NME5, LDHC* compared to normal samples. Further, we checked whether the 5hmC profile is associated with PCa aggressiveness in PR H/L men.

### 5hmC-gene signatures in aggressive tumors

**Figure 4:**
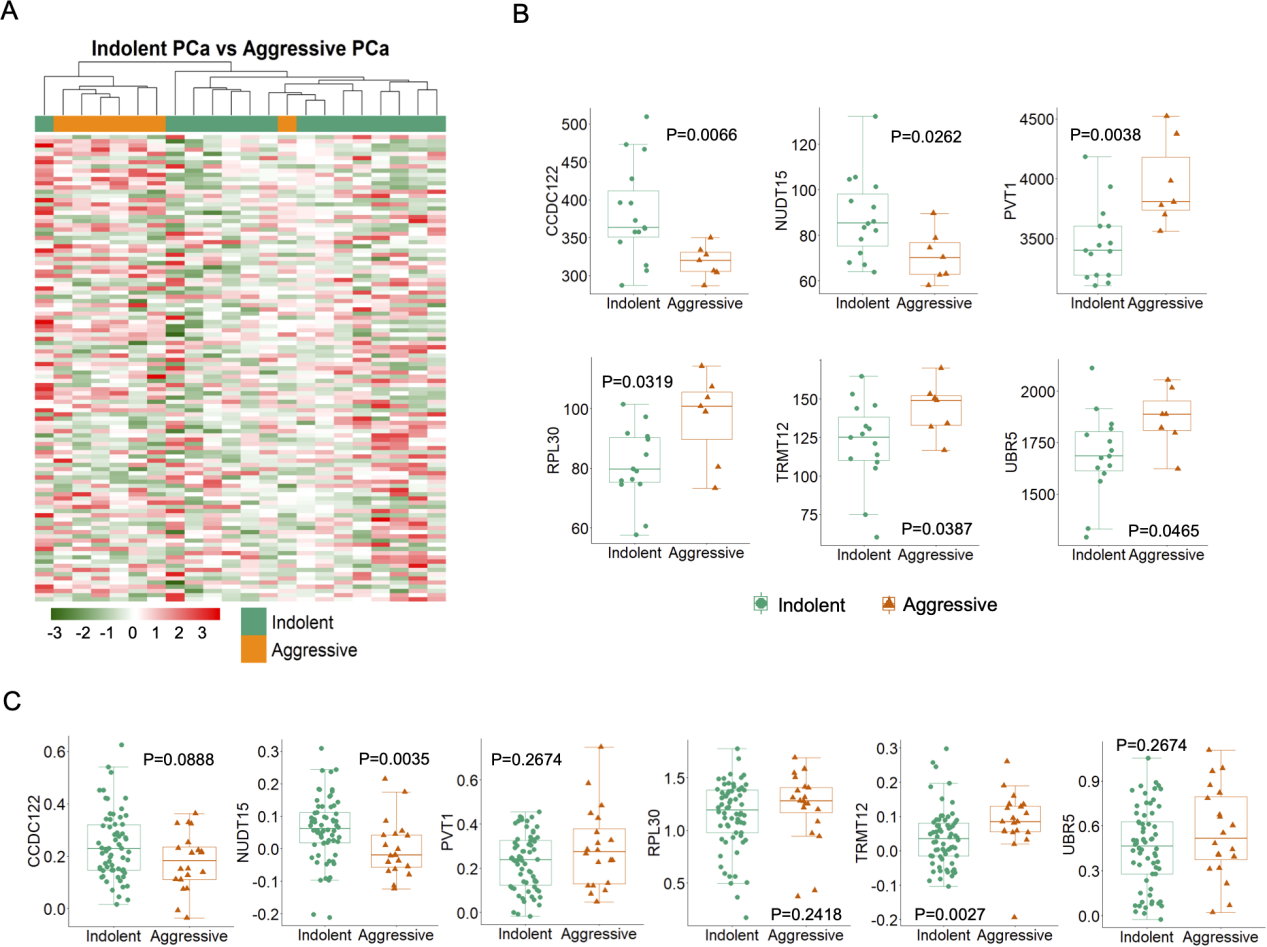
High or low-risk PR H/L PCa patients demonstrated significantly different (P<0.05) and concordant 5hmC-GE signatures. **(A)** The heat map shows 111 DMGs in aggressive patients compared to indolent cases. Representative examples of genes showing association of **(B)** 5hmC levels (n=22) and **(C)** GE levels (n=86) with aggressiveness in PR H/L PCa patients. GS was used to define the risk category of each case. Low risk, GS=<6 & 3 + 4; High risk, GS=4 + 3 & >8.

Based on the Gleason score, we classified as 7 aggressive tumors and 15 indolent tumors in 22 tumor samples from PR H/L men. To detect DMGs associated with high-risk aggressive tumors, we evaluated the difference in 5hmC levels between the two groups and identified 111 DMGs (P<0.05) (**Figure 4A**). Among those DMGs, the most noticeable genes included hypomethylated genes (*CCDC122, NUDT15, BCCIP*, and *KLK10*) and hypermethylated genes (*PVT1, TRMT12, RPL30, UBR5*, *COX6C, ARMC2*) in aggressive PCa patients (**Supplementary Table 4A**). These genes were previously reported for their role in aggressive PCa biology. To check the functional implication of these 111 DMGs, we examined whether their 5hmC levels were positively correlated with their transcriptomic levels. Out of these 111 DMGs, we confirmed 5hmC hypomethylated genes (*CCDC122*, P=0.089 and *NUDT15*, P=0.004) and hypermethylated genes (*TRMT12*, P=0.003; *PVT1*, P=0.267, *RPL30*, P=0.24 and *UBR5*, P=0.27) with same direction GE levels in PR H/L PCa patients (n=86) (**Figures 4B, C**, **Supplementary Tables 4A, B**). These candidate genes in aggressive tumors reveal their significance as potential biomarkers or targets in aggressive PCa patients of PR H/L origin.

### Association of 5hmC levels with poor progression-free survival

**Figure 5:**
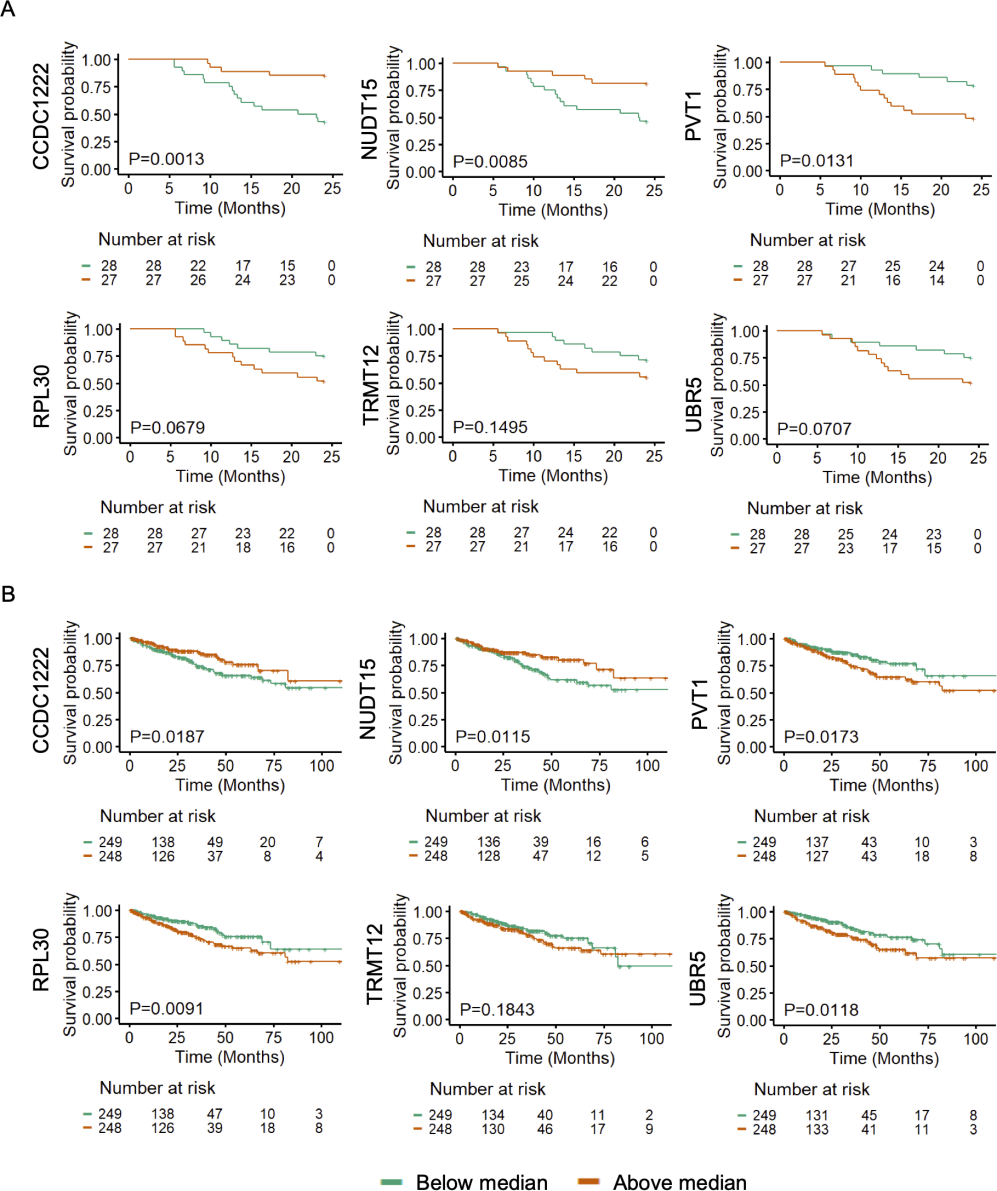
5hmC and GE levels of *CCDC122*, *NUDT15* (low), and *PVT1, TRMT12, RPL30, UBR5* **(high)** are significantly (P<0.05) associated with poor PFS in PCa patients. Representative examples of genes showing association of **(A)** 5hmC levels in cfDNA (n=55) and **(B)** GE levels in tumor tissues (n=497) with poor PFS in PCa patients of mixed origin. The independent cohorts of PCa patients were used for this analysis. Gene expression levels were retrieved from TCGA database (n=497).

The concordant 5hmC and gene expression signature in *CCDC122, NUDT15, TRMT12, PVT1, RPL30*, and *UBR5* may be responsible for poor survival in PCa patients. However, we could not gather clinical follow-up survival data for PR H/L PCa patients. Hence, we examined the association of these genes with survival in PCa patients of mixed origin. We have used the 5hmC data generated from cfDNA for another study for survival analysis (37). We found that lower levels of *CCDC122* and *NUDT15* and higher levels of *PVT1*, *RPL30*, *TRMT12*, and *UBR5* were significantly associated or trending towards significance while associated with poor PFS in PCa of mixed origin (**Figure 5A**, **Supplementary Table 5A**). The consistent findings across diverse DNA sources (tissue biopsy and cfDNA) from PCa patients affirm the significance of altered methylation levels in these genes as reliable indicators for predicting a worse prognosis.

Further, we aimed to investigate whether transcriptomic levels of these genes are also associated with poor PFS in PCa patients. We examined gene expression and survival data from TCGA PCa database (n=497). We found that lower GE levels of *CCDC122* and *NUDT15*, and higher GE levels of *PVT1*, *RPL30, TRMT12*, and *UBR5* showed a clear trend of association with poor prognosis in PCa patients (**Figure 5B**, **Supplementary Table 5B**). Our findings revealed a uniform directional cfDNA 5hmC and tissue GE signature of *CCDC122, NUDT15, PVT1, RPL30, TRMT12*, and *URB5*. These genes are associated with poor survival in mixed PCa populations. This further solidifies the role of these candidate genes in aggressive PCa biology.

## Discussion

Despite high PCa-specific mortality, Puerto Rican Hispanic/Latino (PR H/L) men remained an understudied population (3). Although PCa is slowly growing, around 20-30% of cases show an aggressive phenotype potentially leading to metastasis and poor survival outcomes. Considering PCa racial and ethnic health disparities, we aim to investigate 5hmC changes in PR H/L PCa patients and their role in the aggressiveness of the disease. Our analysis revealed 59 genes having the same direction of epigenetic and transcriptomic changes in tumor tissue of PR H/L men. Further, we found 111 DMGs associated with PCa aggressiveness with six candidate genes having concordant epigenetic-transcriptomics signatures associated with PCa aggressiveness in PR H/L men. Finally, we demonstrated these candidate genes’ 5hmC and gene expression levels for their association with poor PFS in PCa patients. Our findings provide an essential insight into the epigenetic landscape of PCa in the PR H/L patient population. Some of the genes identified in this study are associated with various cancers including PCa, and affect multiple biological processes, such as immune pathways, cell signaling, metabolism, DNA repair, proliferation, and cell cycle (**Supplementary Table 2**).

Our data indicates significant alterations in the 5hmC profile in PCa tissues compared to adjacent normal tissues. The hypermethylated genes include androgen-regulated gene (*AGR3*), and the PCa proliferation-related gene (*RHPN2*). The hypomethylated genes include tumor suppressor genes (*BTG2*, *DKK3* and *PRDM8*), transcriptional repressor (*HIC1, IRF2BP1*), apoptosis related gene (*MAPK7*) and methyltransferases (*PRDM8. PRDM16, PRDM13, KMT5C, FAM86B2, TRMT61A*). *AGR3* overexpressed in PCa tissues vs benign prostate tissues and cancer/benign tissues vs tissues obtained following castration, meaning that androgens regulate these genes and are potentially involved in prostate carcinogenesis (40). It is also reported that *AGR3* is responsible for the activation of the Wnt/β-catenin pathway in human colorectal cancer cells, which is an important mechanism for stemness and cell proliferation in cancer cells (41). Mainly, *AGR3* potentially acts as a negative regulator of AR, inhibiting the activation of genes crucial for controlling prostate cancer growth. In co-cultured cell line experiments, a previous study demonstrated that *RHPN2*, a miR-205 target, positively regulates PCa cell proliferation, invasion, and migration (42).

*BTG2*, a tumor suppressor gene upregulated by *PTEN* and *p53*, was lower in human bladder cancer tissues than normal bladder samples (43). *DKK3* has a protective role in PCa as reported in prostate cell lines (44) and it negatively regulates Wnt/β-catenin signaling pathway (45). Hypomethylation of tumor suppressor genes, such as *BTG2* and *DKK3*, in the tumor group results in decreased gene expression, contributing to prostate cancer proliferation. *HIC1* loss promotes PCa metastasis by triggering epithelial-mesenchymal transition as reported in PCa cell lines, human PCa tissues, and animal model systems (46). In cell line study, *HIC1* also attenuates Wnt signaling, impacting the activation of genes regulated by the canonical Wnt/β-catenin pathway (47)*. IRF2BP1* is a transcriptional corepressor that belongs to the IRF2BP protein family (IRF2BP1, IRF2BP2, and EAP1), and *EAP1* has been reported as a novel AR coregulator in a PCa cell line study (48). Overall, our results suggest key mechanisms involved in PCa development in this high-risk subgroup, including upregulated signatures of negative AR regulation through *AGR3* hypermethylation and *IRF2BP1*-*EAP1* hypomethylation, downregulation of tumor suppressor genes (*BTG2* and *DKK3*), and activation of the Wnt/β-catenin pathway via *AGR3* hypermethylation and *DKK3*, *HIC1* hypomethylation. Multiple pathways, such as DNA damage sensor activity, DNA recombination, and cell cycle-related pathways, were upregulated in tumor samples compared to adjacent normal tissues. These epigenetic modifications can lead to the dysregulation of key tumor suppressor genes and oncogenes, impacting PCa proliferation, metastasis, and treatment response. Aberrant methylation can contribute to the heterogeneity of PCa by altering gene expression patterns, impacting disease aggressiveness and therapeutic resistance, ultimately affecting overall prognosis and survival in this high-risk subgroup.

The DMGs from our findings also overlapped with a previous 5hmC study (8) on PCa and gene expression dataset from TCGA. For example, we found *AGR3* and *RHPN2* hypermethylation and *BTG2* and *MAPK7* hypomethylation in both 5hmC datasets. We also found upregulation of *AGR3* and downregulation of *DKK3, PRDM8*, and *TP53AIP1* in our 5hmC study and TCGA gene expression dataset. Interestingly, the PR H/L cohort also showed unique differentially methylated genes, including hypomethylated genes such as *IRF2BP1*, *HIC1*, *MAPK11* and hypermethylated genes such as *CDC25C*, *FLT3*, *NME5, LDHC* (**Supplementary Table 3C**). These identified gene signatures in PR H/L PCa tumors are highly associated with AR regulation. Downregulation of apoptotic and tumor suppressor pathways leads to prostate cancer aggression, proliferation, survival, and therapeutic resistance.

A previous study on PCa patients, showed that tumor aggressiveness is associated with dysregulation of gene expression in prostate cancer (49). We also demonstrated that hypomethylated genes (*CCDC122*, *NUDT15*) and hypermethylated genes (*PVT1*, *RPL30*, *TRMT12*, and *UBR5*) have concordant gene expression changes in PR H/L PCa patients. *CCDC122* and *NUDT15* are located on the nearby cytogenetic band of 13q14.11 and 13q14.2, respectively, and deletion of both genes is associated with PCa growth and survival (50, 51). Notably, allelic loss at 13q14 has been reported in 33% of human prostate tumors (52, 53) and associated with high prostate tumor grade and stage (54, 55). We believe that hypomethylated 5hmC and downregulated expression of these genes in our study indicate that they could be critical 5hmC markers of PCa in PR H/L men.

We showed that PVT1, *RPL30*, *TRMT12*, and *UBR5* are 5hmC hypermethylated and overexpressed in aggressive tumors. Previous studies showed that these genes are important for AR regulation, PCa growth and aggressiveness. *PVT1* is located on the 8q24 along with c-Myc which well-reported site for copy number gains in different cancers (56). Previously*, PVT1* promoter 5mC hypomethylation was found to be associated with worse prognosis in renal cell cancer due to *PVT1*-*MYC* upregulation (57). *RPL30* (8q22.2), an overexpressed ribosomal proteins (RPs) in human PCa tissues (58), is positively correlated with co-amplification of 8q22-24 regions containing genes encoding the Myc-PVT1 (8q24.21) (59, 60). *TRMT12* (8q24.13), the tRNA methyltransferase, showed strong binding by the AR in castrate-resistant PCa (CRPC) tissues and was overexpressed in CRPC tissues compared to benign or untreated prostate tissues (61). UBR5 (8q22.3) was reported as a top PCa-related E3 ubiquitin ligase in PCa tumors which is strongly associated with PC progression and aggressiveness (62). In primary PCa biopsies, it has been demonstrated that chromosome 8q gain is correlated with early progression in hormonal-treated PCa (63). In our study, *PVT1*, *TRMT12*, *RPL30*, and *UBR5* hypermethylation demonstrates that, in addition to 8q gain, 5hmC-based methylation and the corresponding increase in gene expression levels are important mechanisms for the higher activity of these genes in PR H/L men with aggressive PCa.

Further, we tested six genes (*CCDC122, NUDT15, PVT1, TRMT12, RPL30*, and *UBR5*) in different cohorts of PCa patients from mixed origin and demonstrated their 5hmC and gene expression levels are associated with poor progression-free survival. Here, the 5hmC levels were measured in cfDNA, which shows that these gene methylation levels can be prominently detected in blood and can be used to predict survival outcomes. The results of this analysis bolster our findings in PR H/L men that these six genes are associated with PCa aggressiveness and, hence, with poor PFS.

The congruent directional changes in 5hmC and gene expression could be critical in aggressive PCa biology; hence, validating them as potential biomarkers and therapeutical targets for aggressive PCa among PR H/L men is worthwhile. The novelty of our study is identifying the 5hmC candidate genes and understanding the potential role of 5hmC in an understudied PR H/L population with PCa. As far as we know, this is the first report studying 5hmC methylation in this Puerto Rican population. However, our study has some limitations, which include a small sample size and the unavailability of tumor-normal gene expression data. Due to this, we were not able to directly determine associations with transcriptomic levels of 628 unique DMGs compared to normal tissue in this population. Our ability to perform survival analysis in PR H/L men was also restricted due to the unavailability of clinical follow-up data. In the future, it is important to extend this analysis to a larger cohort with follow-up information and tumor-normal transcriptomics data to identify exclusive genes associated with high specific mortality in PR H/L men with PCa.

In conclusion, our study identifies important gene signatures in RP H/L men with PCa (**Supplementary Table 6**) and demonstrates that *CCDC122, NUDT15, PVT1, TRMT12, RPL30*, and *UBR5* are associated with PCa aggressiveness in PR H/L men, hence, poor survival outcomes. The development of biomarkers for PCa aggressiveness will provide more effective tools for the diagnosis of clinically significant disease and facilitate the selection of potential therapeutical drug targets.

## Data Availability

The original contributions presented in the study are publicly available. This data can be found here: https://www.ncbi.nlm.nih.gov/geo/; GSE293385.
